# Ovarian response to controlled stimulation and its predictors in a limited-resource setting

**DOI:** 10.1186/s12905-024-02991-7

**Published:** 2024-05-07

**Authors:** Munira Dermolo, Meseret Ansa, Melkamu Siferih

**Affiliations:** 1 Department of Obstetrics and Gynecology, St.Paul’s Hospital Millennium Medical College, Addis Ababa, Ethiopia; 2 Reproductive Endocrinology and Infertility, Department of Obstetrics and Gynecology, St.Paul’s Hospital Millennium Medical College, Addis Ababa, Ethiopia; 3 Department Obstetrics and Gynecology, School of Medicine, Debremarkos University, Debremarkos, Amhara Ethiopia

**Keywords:** Ovarian response, Predictors, Controlled stimulation, Ethiopia

## Abstract

**Background:**

Infertility remains a serious health concern for Ethiopian women. Most of its treatment approaches entail controlled ovarian stimulation, the responses of which vary. However, there are no data on ovarian response to stimulation or its predictors in our situation. Thus, the current study aimed to assess the ovarian response to controlled stimulation and identify predictors.

**Methods:**

A retrospective follow-up study was undertaken from April 1, 2021, to March 31, 2022, among patients who had first-cycle controlled ovarian stimulation at St.Paul’s Hospital Fertility Center in Addis Ababa, Ethiopia. Clinical data were extracted using a checklist. SPSS-26 for data analysis and Epidata-4.2 for data entry were employed. The binary logistic regression model was fitted. A p-value < 0.05 indicated a significant association. The ROC curve was used to determine cutoff values and identify accurate predictors.

**Results:**

A total of 412 study participants were included in the final analysis. The patients had a mean age of 32.3 ± 5.1 years (range: 20 − 4). The good ovarian response rate was 67% (95% CI: 62.2–71.5). An anti-Mullerian hormone (AMH) concentration < 1.2ng/ml (AOR = 0.19, 95% CI (0.06–0.57)), an antral follicle count (AFC) < 5 (AOR = 0.16, 95% CI (0.05–0.56)), and an induction length < 10 days (AOR = 0.23, 95% CI (0.06–0.93)) were significantly associated with ovarian response. The prediction accuracies for the AFC and AMH concentrations were 0.844 and 0.719, respectively. The optimal cutoff point for prediction was 5.5 AFC, which had a sensitivity of 77.2% and a specificity of 72.8%. However, its positive and negative predictive values were 85.2% and 61.1%, respectively. For AMH, the optimal cutoff value was 0.71ng/mL, with a corresponding sensitivity and specificity of 65.2% and 66%. At this value, the positive and negative predictive values were 63.8% and 67.3%, respectively.

**Conclusion:**

Only two-thirds of our patients achieved a good ovarian response. Induction duration, AMH concentration, and AFC were found to be predictors, with the AFC being the strongest predictor. Therefore, the AFC should be performed on all of our patients, and the AMH is selectively employed. Future research must verify the best cutoff points and investigate additional factors affecting ovarian response.

## Background

Infertility was defined as the inability to conceive after a year of consistent, unprotected sexual activity because of either the individual or the partner’s decreased fertility [1]. Infertility impacts one-fourth of couples in developing countries [2, 3]. After the age of 30, women’s fecundity declines due to a proportionate decrease in the quantity of eggs that are accessible [4]. Nonetheless, it is challenging to predict the rate at which a given woman’s reproductive function deteriorates [5]. Interindividual variations exist among women who suffer from infertility [6]. Ensuring that the patient has enough eggs to develop high-quality embryos for implantation into the uterus without running the danger of overstimulating the ovaries is one factor that contributes to the success of in vitro fertilzation (IVF) [7].

The goal of ovarian stimulation medication therapy is to promote the growth of ovarian follicles [1, 8]. The administration of exogenous gonadotropin, which enables the collection of several oocytes in a single cycle, forms the cornerstone of ovarian stimulation for assisted reproduction. So-called controlled ovarian stimulation (COS) is employed when these medications are given only to stimulate follicles and oocyte collection is finished with assisted reproductive technology (ART) [9]. Patients of the same age, following the same protocol, or using the same medication all have quite different responses to controlled ovarian stimulation [6, 7, 10]. While studies [11–24] have shown that only a handful of women who are receiving controlled ovarian stimulation (2–50%) have a poor ovarian response, there is no consensus on what constitutes a good or poor ovarian response. However, a single stimulation cycle is necessary to predict poor ovarian response [25].

Many factors such as patient age, premenstrual basal concentrations of hormone markers (e.g., FSH, LH, estradiol, inhibin B, and more recently AMH), and ultrasound measurements (pretreatment antral follicle count) are predictive of ovarian response [6, 7, 9, 10]. However, given the high expense and difficulty of therapy for treatment-seeking couples, identifying ideal markers of ovarian response is very useful for helping reproductive specialists choose the best dosage of gonadotropins for ovarian stimulation and for predicting the outcomes of ART [9, 10, 26, 27].

One of the biggest health issues facing Ethiopian women today is infertility [3]. Furthermore, the fact that Ethiopia has only one government-run fertility institution complicates the issue’s resolution. To the best of our knowledge, there are no data about the ovarian response or its predictors.

It is crucial to identify patients based on their ovarian response to modify treatment protocols, customize care for patients, prevent treatment failures and complications, and improve pregnancy outcomes [6, 8].

Thus, the present study aimed to determine the degree of ovarian response to controlled stimulation and identify its predictors at St.Paul’s Hospital Millennium Medical College in the Center for Fertility and Reproductive Medicine by examining clinical characteristics, treatment protocols, gonadotropins, hormonal factors, and transvaginal ultrasound data.

## Methods

### Study setting, design, participants

A retrospective follow-up study was undertaken from April 1, 2021, to March 31, 2022, at St.Paul’s Hospital Millennium Medical College Reproductive Medicine and Fertility Center in Addis Ababa, Ethiopia. It is the first public health facility to provide advanced options for infertility treatment such as in vitro fertilization (IVF). Established in January 2019, the Center for Fertility provides annual infertility treatment to thousands of couples [28]. The center offers several work-ups for infertility, such as hysterosalpingography, semen analysis, hormone analysis including anti-Mullerian hormone concentration, and transvaginal antral follicular count. Despite the center’s establishment having occurred three years prior, the study period was purposefully chosen because of the increased accessibility of investigative modalities, medications including gonadotropins, and infertility experts. The period of data collection was April 1, 2022–April 30, 2022.

The women who visited the center for infertility treatment composed the source population. The study population consisted of all the women who met the inclusion criteria during the study period and participated in the first cycle of controlled ovarian stimulation. To be included in the final analysis, all relevant variables were needed, such as the determination of the oocyte status at retrieval and the antral follicular count. Women with incomplete documentation and a single ovary were excluded. Those with high and normal responses were regarded as having adequate responses.

On the third day of the menstrual cycle, the basal concentrations of FSH, estradiol (E2), and AMH at any point during the cycle were determined. From day three to day five of the same cycle, all patients underwent ovarian ultrasonography handled by fertility specialists utilizing two-dimensional transvaginal ultrasound with a transvaginal probe operating at 5–10 MHz. For the AFC and AMH levels, the standard normal were ≥ 5 counts and ≥ 1.2 ng/ml, respectively [29, 30].

The three primary stimulation protocols used were antagonistic, minimal-stimulation, and long protocols. Patients under 35 years old with good ovarian reserve (the AFC greater than 5) are typically candidates for the long protocol. After confirming the existence of a cyst or dominant follicle, the patient was prescribed 3.6 mg of goserelin (Zoladex) subcutaneously on day 21 of her period. She returned for stimulation on day 2 of her period or 14 days after receiving the Zoladex injection, whichever came first. Human menopausal gonadotropin (HMG) is started at a dose determined by age and body mass index (BMI) and can be used either alone (Menopur) or in combination with recombinant FSH (Gonal-F) if there are no contraindications to stimulation (no ovarian cysts larger than 10 mm). Transvaginal ultrasonography was used to observe changes in the endometrium and follicle size, and the dosage was adjusted according to the findings. Patients older than 35 years, with low ovarian reserve (AFC < 5), and who could not afford a lengthy treatment course were advised to receive minimal stimulation. Starting on day 2 of the cycle, 5 mg of letrozole was taken orally for 5 days, and 150 IU or 225 IU of hMG SC was added on day 4. Cetrotide-mediated downregulation commenced when the size of the leading follicle reached 14 mm. When minimal stimulation failed or the desired response was not as expected, antagonist protocols were frequently used. The gonadotropin dose is not fixed and is instead determined by age and BMI. This is the only distinction between the minimally stimulated regimen and the antagonist regimen. The minimally stimulated regimen started with letrozole on day 2 in contrast to the antagonist regimen.

### Sample size determination and sampling technique

The sample size was calculated to be 384 using the single population proportion formula, with a 95% confidence interval, 5% margin of error, and 50% proportion of good ovarian response because the degree of ovarian response in the study region was unknown. By accounting for a 10% rate of inadequate documentation, the ultimate sample size became 422. To choose the sampling units, convenience sampling was used, which involved using the medical record number found in both the chart and computerized medical records.

### Operational definitions

The ovarian response (as good or poor) was defined as the degree to which the ovarian follicles developed in response to gonadotropins. On the day of the oocyte maturation trigger, > 3 follicles and/or > 3 oocytes retrieved were considered to indicate a good response. A poor ovarian response was the reverse of what happens in a good ovarian response [9, 12, 31]. The identified ovulatory factors included hyperprolactinemia, polycystic ovary syndrome (PCOS), and thyroid disorders. Intrauterine adhesions, bicornuate uterus, polyps, and submucous myoma were considered uterine/endometrial factors.

### Study variables

The degree of ovarian response was the dependent variable. The independent variables included age, the duration of infertility, the cause of infertility, the stimulation protocol employed, the induction length, the type of medication, the antral follicular count (AFC), hormonal test results for follicle-stimulating hormone (FSH), thyroid-stimulating hormone (TSH), estradiol, prolactin, and anti-Mullerian hormone (AMH).

### Data collection tools, procedures, and data quality assurance

A structured checklist was used to collect the patient data. It was developed using registration log books and various literature [6, 7, 10–12] to address the sociodemographic characteristics, hormonal test results, sonographic findings, treatment protocols, and types of medications used. One day of training was given to data collectors and supervisors. The principal investigator provided the training. Two certified nurses with at least two years of experience at the center worked as data collectors, while two general practitioners served as supervisors. The quality of the data was evaluated each day after data collection.

### Data processing and statistical analysis

The data were coded, verified for accuracy, and entered into Epidata version 4.2. Then, SPSS version 26.0 was subsequently used to export and analyze the data. One-way ANOVA, post hoc tests, and independent samples t-tests were used for evaluating relationships and differences between significant categorical factors and between those variables and the continuous variable. The data were summarized, tabulated, analyzed, and expressed with descriptive statistics such as frequency, percentage, mean, median, and interquartile range. Frequencies and percentages are presented for categorical variables, the mean is presented for continuous variables with normally distributed data, and the median and the interquartile range are presented for data with a nonnormal distribution. To identify predictive factors of ovarian response, a binary logistic regression model was applied. The final model of multivariable analysis included all variables with a p-value of less than 0.25 in bivariate analyses. Stepwise variable selection was employed. Model fitness was checked by the Hosmer-Lemeshow test and goodness of fit by the omnibus test of coefficients. The adjusted odds ratio was used to determine the strength of the measure of association. A p-value < 0.05 was considered to indicate statistical significance in the final model. ROC curve analysis was used to determine the ideal cutoff points for the selected predictors and to assess the predictive accuracy, sensitivity, and specificity of the predictors of ovarian response.

## Results

### Sociodemographic and clinical characteristics

Four hundred and twelve patient charts and electronic medical records were reviewed and included in the final analysis. The mean age of the participants was 32.3 ± 5.1 years (mean ± SD), and the age range was 20 to 47 years. More than half (61.4%) of the participants were under 35 years old. Among the patients, 93.2% lived in urban areas and nearly all (98.3%) were married. In addition, the majority (70.6%) of the study participants was parous, and 44.6% had at least a secondary level of education. Regarding the causes of infertility, the tubal factor was responsible for half (50.2%) of the cases. Polycystic ovarian syndrome (PCOS) was present in four patients (1%) based on the Rotterdam Criteria. Most of the patients (54.6%) were not able to conceive for more than five years (Table 1). Three patients with ovarian hyperstimulation syndrome were diagnosed and treated by infertility specialists because the study included patients with PCOS and other hyperresponders.

**Table 1:** Sociodemographic characteristics of participants at St.Paul’s Hospital (April 1, 2021–March 31, 2022)

Characteristics	Category	(*N* = 412)(n, %)
Age	< 35	253(61.4)
	≥35	159(38.6)
Address	rural	28(6.8)
	urban	384(93.2)
Religion	Orthodox	235(57.0)
	Muslim	128(31.1)
	Protestant	28(11.9)
Occupation	housewife	126(30.5)
	civil servant	151(36.7)
	merchant	135(32.8)
Marital status	married	405(98.3)
	single	7(1.7)
Educational status	no formal education	12(2.9)
	primary School	58(14.1)
	secondary School	184(44.6)
	tertiary level	158(38.4)
Parity	nulliparous	121(29.4)
	parous	291(70.6)
Causes of Infertility	Tubal factor	207(50.2)
	Male factor	95 (23.1)
	Both female and male factor	9 (2.2)
	Unexplained	87(21.1)
	Ovulatory factors	7(1.7)
	Endometrial/uterine factors (treated)	7(1.7)
Duration of Infertility	< 5 years	187(45.4)
	≥ 5 years	225(54.6)

### Results of hormonal testing and transvaginal ovarian sonography

FSH was determined in 63.3% of patients with a median value of 6.6 (IQR: 4.37–9.06), AMH in 23.3% of women with a median value of 0.7 (IQR: 0.28–1.58), AFC in all patients with a median value of 6.9 (IQR: 4.36–12.36). On the other hand, the median values of TSH (in miu/l), prolactin (in ng/ml), and estradiol (in pmol/l) were 1.7 (1.13–2.7), 17.15 (11.96–23.74), and 56 (36.65–133.7), respectively. Of the women whose FSH levels were checked, 2.7% had abnormal values; 67.7% had AMH levels less than 1.2; 39.1% had Antral follicle counts (AFCs) less than 5 (Table 2).

**Table 2:** Hormone test results and the antral follicle counts

Results	Category	n(%)
FSH (*n* = 261) in IU/l	0–5	93(35.6)
	5–20	161(61.7)
	> 20	7(2.7)
AMH (*n* = 96) in ng/ml	< 1.2	65(67.7)
	≥ 1.2	31(32.3)
AFC (*n* = 412)	< 5	162(39.3)
	≥ 5	250(60.7)
TSH (*n* = 284) in miu/l	0-0.4	14(4.9)
	0.4–4.5	247(87.0)
	> 4.5	23(8.1)
Prolactin (*n* = 292) in ng/ml	< 35	269(65.3)
	≥ 35	23(5.6)

### Stimulation protocol and the ovarian response to controlled ovarian stimulation

For the stimulation protocol that was employed, 62.7% of the participants used Ministim. Conversely, 8.7% and 28.6% of the patients, respectively, followed the antagonist and long protocols. The stimulation lasted an average of ten days (minimum four days, maximum fifteen days). A good ovarian response (meaning that more than three oocytes were retrieved) of 67% (95% CI (62.2–71.5)) was achieved. A mean of 7.6 ± 7.5 (0–60) oocytes were retrieved. Using one-way ANOVA and post hoc tests, this study also attempted to examine the mean difference in the total number of oocytes retrieved for each patient between treatment protocols. The mean number of oocytes retrieved across all treatment protocols varied significantly (F.Statistics = 101.8 with p-value < 0.001). For each of the three treatment protocols, a difference was noted (post hoc Bonferroni p-value < 0.001). To determine whether there was a mean difference in the total number of oocytes retrieved between the two regularly utilized medication types (pure FSH and combined form (HMG)), the independent samples t-test was also employed. A mean difference = 5.4, 95% CI (3.19–7.9), and p-value < 0.001 indicated a statistically significant difference.

### Predictors for ovarian response after controlled stimulation

The following twelve factors were subjected to univariate analysis: patient age, duration of infertility, cause of infertility, type of protocol used, induction length in days, type of medication, AFC, FSH, TSH, level of estradiol, level of prolactin, and AMH. Except for the cause of infertility, eleven factors had a p-value of < 0.25. The variance inflation factor was used to verify the multicollinearity test (mean VIF = 1.1). The model with the lowest Akaike information criterion and the lowest Bayesian information criterion (AIC = 346.2, BIC = 339.5) included seven variables. Anti-Mullerian hormone levels, antral follicular count, and duration of stimulation were found to be predictors of ovarian response to controlled stimulation with a p-value < 0.05. The Hosmer-Lemeshow test, which was used to evaluate the goodness of fit test, was not significant (p-value = 0.508), while the omnibus test, which assessed model fitness, had a p-value < 0.001.

Keeping the other variables constant, compared to patients with a normal range of Anti-Mullerian hormone levels, those with an AMH level < 1.2 had an 81% reduced ovarian response to controlled ovarian stimulation (p-value = 0.0003). Similarly, patients with an AFC of less than five antral follicle counts had 84% lower odds of ovarian response than patients with higher AFC did (p-value = 0.0004), after adjusting for other confounders. Finally, when all the other variables were held constant, more than ten days of induction increased the chance of a good ovarian response by 77% (p-value = 0.04) (Table 3).

**Table 3:** Predictors for ovarian response to controlled stimulation (*n* = 412)

Variables	Category	Ovarian response		AOR (95% CI)
		Good response	Poor response	
Age	< 35	191	62	0.64(0.22–1.90)
	≥ 35	85	74	1
Duration of infertility	< 5	128	59	1.31(0.44–3.91)
	≥ 5	148	77	1
AMH **(n = 96)**	< 1.2	22	43	0.19(0.06–0.57) **
	≥ 1.2	24	7	1
AFC	< 5	63	99	0.16(0.05–0.56) **
	≥ 5	213	37	1
Stimulation protocol	Ministim	139	119	0.46(0.32–0.54)
	Long protocol	115	3	0.39(0.07–2.20)
	Antagonist	22	14	1
Induction length (days)	< 10	197	119	0.23(0.06–0.93)*
	≥ 10	79	17	1
Medication used	Pure FSH	34	10	0.51(0.07–3.56)
	Combined(HMG)	242	126	1

### Receiver operating characteristics (ROC) curve analysis

**Fig. 1:**
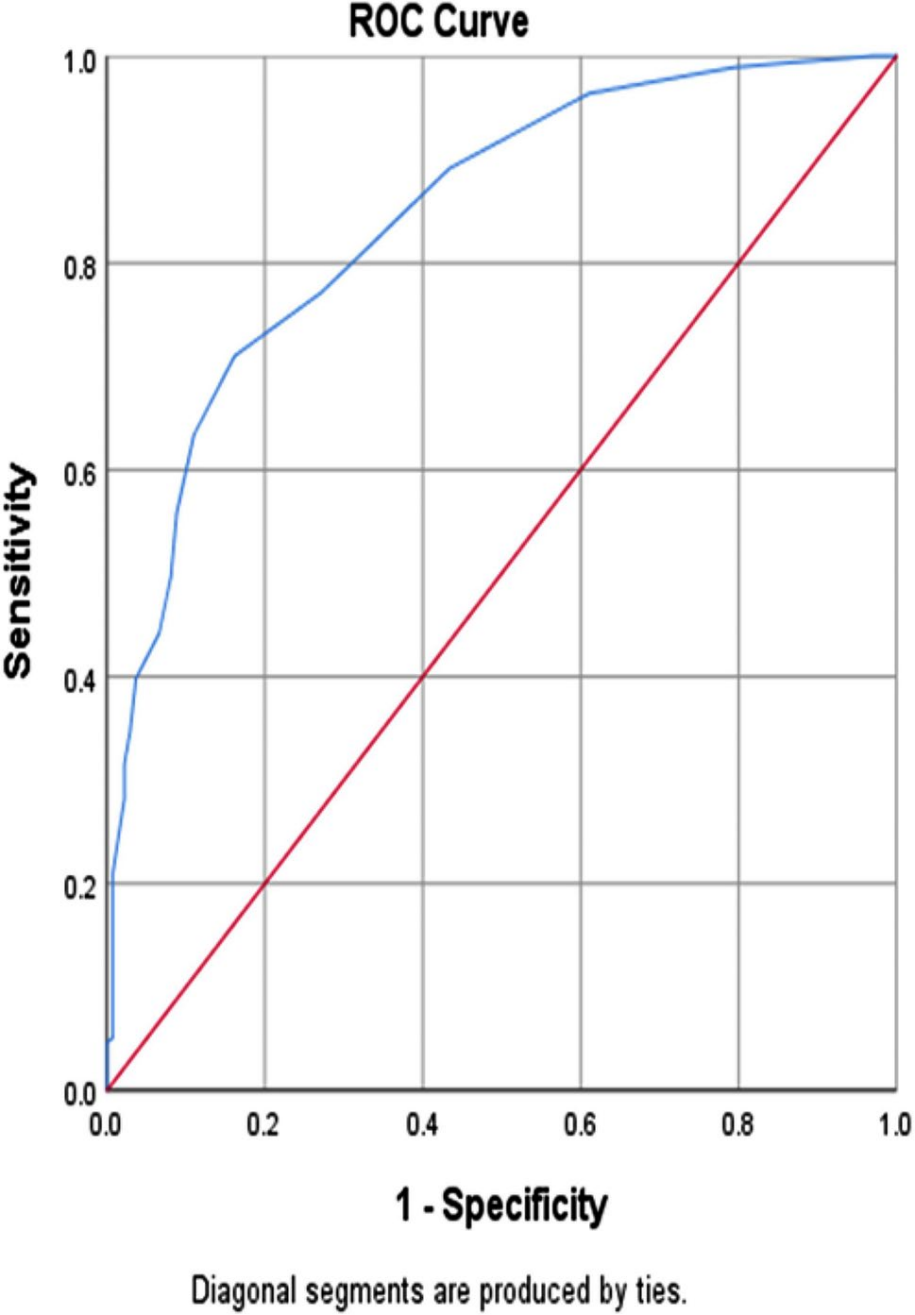
ROC curve for the antral follicular count

ROC analysis was performed to determine the predictive accuracy of the predictors. The ROC curve for the AFC is as follows: AUC = 0.844, 95% CI (0.805, 0.883), and p-value < 0.001. According to the recently introduced best approach for determining the cutoff point, the index of the union selection method, the ideal cutoff level for discriminating between good and poor ovarian response was a follicle count of 5.5; and 72.8% was the specificity and 77.2% was its sensitivity. At this ideal cut-off point, the positive and negative predictive values were 85.2% and 61.1%, respectively (Fig. 1).

**Fig. 2:**
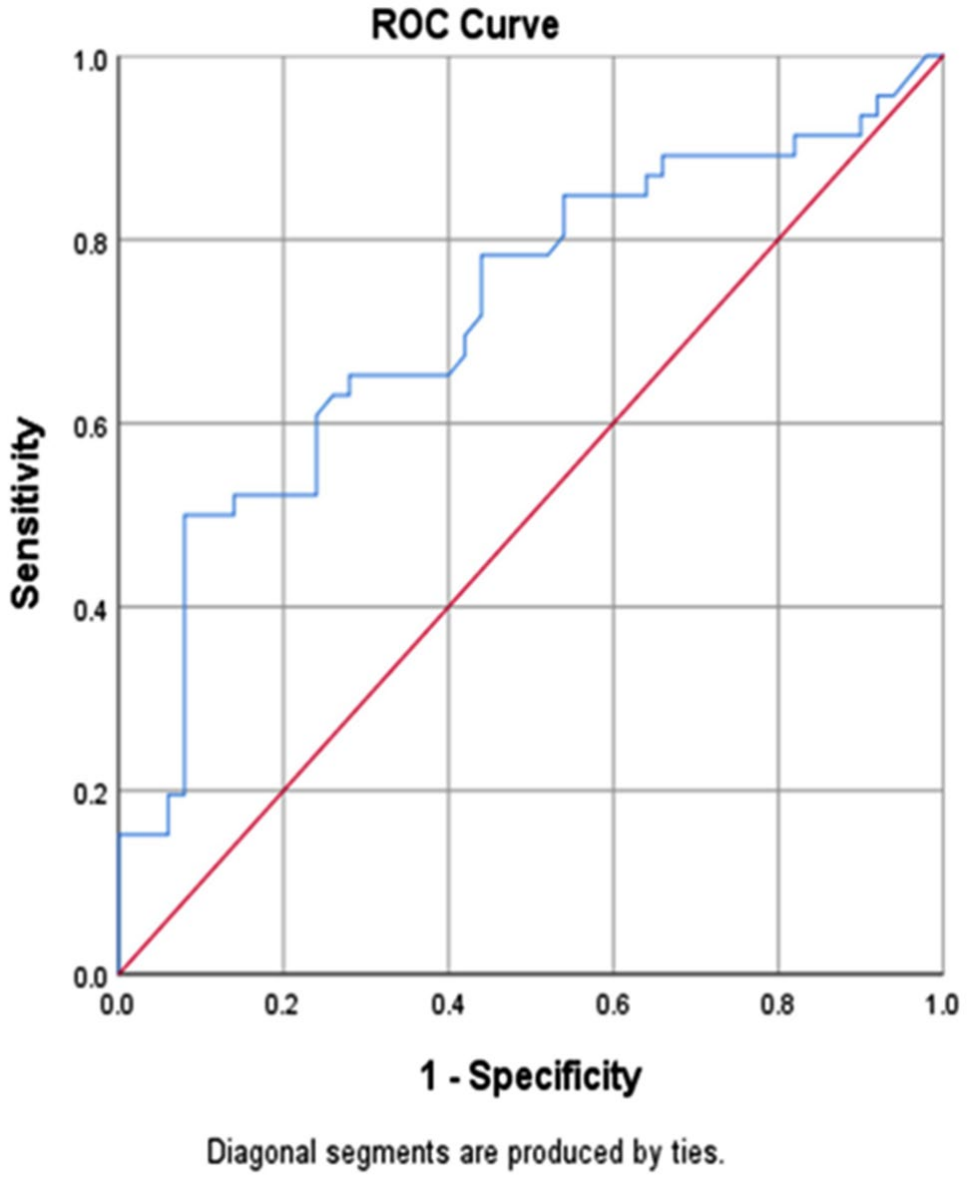
ROC curve for anti-Mullerian hormone concentration

The area under the curve (AUC) for anti-Mullerian hormone (AUC = 0.719, 95% CI (0.615, 0.823), p-value < 0.001) is displayed by the next ROC curve. With a sensitivity and a specificity of 65.2% and 66%, respectively, the discriminative optimum cutoff point for AMH was 0.71ng/ml. At this ideal cutoff point, the positive and negative predictive values were 63.8% and 67.3%, respectively (Fig. 2).

**Fig. 3:**
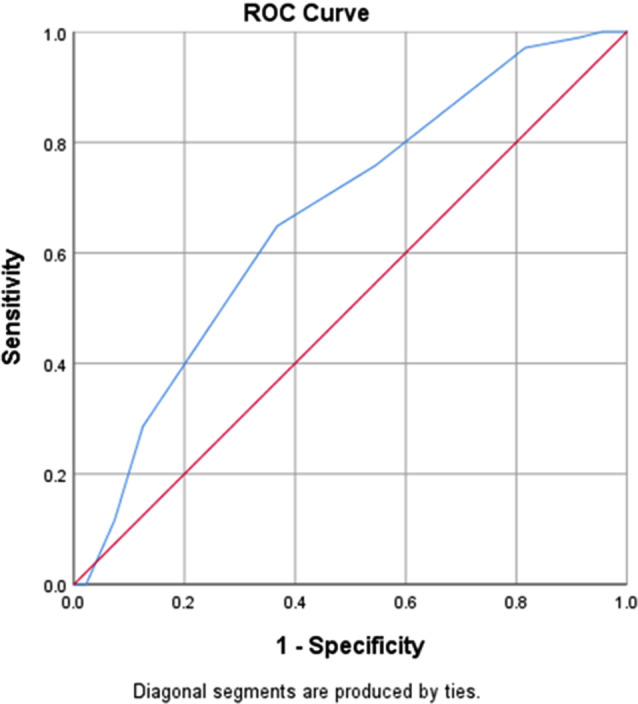
ROC curve for induction length

The third involved analyzing the ROC curve for the induction length. The area under the curve (AUC) was 0.668, 95% CI (0.611, 0.724), and the p-value was < 0.001). Although there was a significant association between induction length and ovarian response, the predictive accuracy was not very strong (AUC < 0.7) (Fig. 3).

**Fig. 4:**
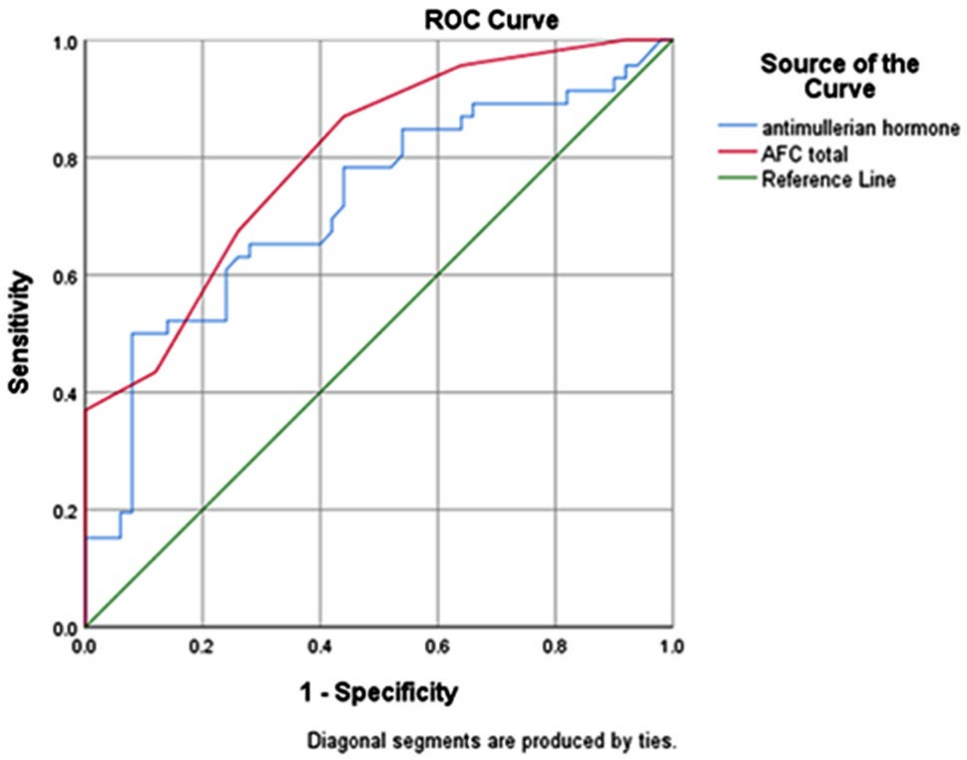
ROC curve for combination testing for both the AFC and AMH concentration

When we evaluated how combined testing improved the predicted accuracy of the two abovementioned good predictors, anti-Mullerian hormone and antral follicular count, the combined test did not significantly improve the prediction of AFC, according to receiver operating characteristics curve analysis (AUC = 0.801, 95% CI (0.715, 0.887), p-value = 0.07) (Fig. 4).

## Discussion

This study was conducted to evaluate the degree of ovarian response and its predictors among women who had first-cycle controlled ovarian stimulation at St.Paul’s Hospital in Addis Ababa, Ethiopia. This is the first study in the nation to evaluate ovarian response through the use of clinical factors, transvaginal ultrasound, gonadotropins, and stimulation protocols. The overall percentage of good ovarian response was 67% according to the number of oocytes retrieved. The ovarian response was found to be predicted by the AFC, AMH concentrations, and induction length.

Two-thirds of our patients achieved a good ovarian response, which was consistent with most previous studies [11, 13, 15–24]. However, compared to studies performed in Egypt (94%) [32] and the United Kingdom (88.9%) [12], this number was much lower. This percentage was, still greater than the 50% registered with the American Society for Reproductive Medicine [14]. The lack of an agreed-upon definition for ovarian response may help to explain this. Instead of quantifying the number of oocytes retrieved, ovarian response has been defined based on factors such as advanced age, cycle cancellation, leading follicles before trigger, follicular growth after trigger, previous poor ovarian response, and abnormal ovarian reserve test results including peak estradiol level [6, 7, 9, 10, 12, 25, 33]. Furthermore, women over the age of 40 and those with endocrine problems such as PCOS or hypo- or hyperthyroidism were included in our study.

Concerning the prognostic value of the AFC and AMH concentration for ovarian response, our findings were in line with earlier research [9, 26, 31, 34–41] that employed various treatment protocols. Compared to conventional indicators such as age, basal FSH, and estradiol, AFC and AMH were superior predictors of ovarian response. These findings will assist clinicians in deciding whether to start gonadotropins based on the AFC and AMH levels as opposed to patient age and baseline FSH levels [12, 40, 42, 43]. A further implication could be heightened absorption of AMH and the AFC in contrast to that of FSH, which might be less indicative of an ovarian response [35, 41]. This, however, contradicts the findings of a systematic review [5], which indicated that all tests, including the AFC and AMH levels, have little clinical significance in terms of predicting poor response. As no test could accurately predict all patients’ poor ovarian response, it was advised that the best course of action would be to start the first cycle of ovulation induction without performing any precycle testing [25].

Unlike prior studies [12, 39, 44–48], which indicated similar predictive values of AFC and AMH, our findings showed that the AFC was a better predictor of retrieval success than was the anti-Mullerian hormone concentration. However, other studies [41, 49, 50] showed that the AMH concentration had a greater predictive value than the AFC. These differences could be explained by variations in transvaginal ultrasound sensitivity, inter- and intraobserver variability in ultrasound examination, variations in the repeatability and cycle stability of AMH, and the use of similar or different stimulation protocols for comparison. The AFC performed better, according to certain studies [10, 51, 52], which was in keeping with our findings. Because AMH levels are expensive and difficult to measure and access, our finding that the AFC has a greater predictive value also has significant repercussions. These findings suggested that both the AFC and AMH concentrations are suitable for assessing ovarian response. Clinicians can readily determine the antral follicle count in infertility patients on a routine basis. Women older than 35 and women with PCOS can benefit from the selective use of AMH when utilizing AFC as a surrogate marker [53]. The absence of standardization in AMH assays and interindividual variability are other drawbacks of using AMH [9].

The optimal thresholds for the AFC and AMH concentration for predicting good or poor ovarian response were 5.5 and 0.71ng/ml, respectively, in our study, in contrast with previous studies (AFC-10, AMH-0.99) [12], and (AFC-8.5, AMH-1.22 [10]. The population variations in the study areas may explain the difference. A further explanation can stem from employing distinct techniques to ascertain ideal threshold values. In contrast to the current study, which used the index of union approach to determine the ideal cutoff point, the majority of earlier studies used the highest sum of sensitivity and specificity. Similarly, there was a correlation between the two important predictors and patient age, indicating that our research did not exclude older age groups. However, these findings were similar to the findings of a Chinese cohort study [54] in which the optimal AMH concentration was 0.8ng/ml, and that of the UK report [55], which was 0.7ng/ml concentration. The application of a single protocol in those two studies was the distinction. In many studies [56–58], the optimal AFC cutoff point was shown to be in the range of 3 to 10.

According to the current evidence, induction length was found to be a predictors of ovarian response. However, we were unable to locate a previous study demonstrating the exact length of stimulation as a predictor of ovarian response. These findings regarding the average length of stimulation were inconsistent with those of studies conducted in Brazil and the UK [12, 59]. Nonetheless, given their tight connection with the nature of the treatment protocol employed, these outcomes made sense. This may also call for an evaluation of the length of the menstrual cycle as a factor affecting the choice of treatment protocol and the ovarian response. Consequently, it is important to take into account treatment protocols and menstrual cycle length when interpreting induction length since this consideration will help individualize care for each patient.

The retrospective nature of this study was one of its main limitations. Advanced age, endometriosis status, inheritance status, endocrine problems, BMI, menstrual cycle duration, hormone therapy status during the past three months, and pelvic surgery were among the factors affecting ovarian response and were not considered in this study. There was no attempt to determine the predictive performance of the AFC and AMH concentration according to the treatment protocols. Due to the use of 2-D ultrasound rather than 3-D ultrasound performed by our setup, comparisons were challenging. The quality of the hormone tests could not be evaluated. Hyperresponders and normal responders were not distinguished in the study. The study did not account for the subsequent induction cycles. The use of ROC curve analysis to display the relative predictive values and ideal cutoff levels for the predictors was one of the study’s strengths. By using electronic medical records, we were able to lower recall bias and increase response rates. Since this was the first exploration in our context, it will provide important preliminary data for future studies. The conclusions of this study should be interpreted carefully in light of these limitations.

## Conclusion

Only two-thirds of our patients achieved a good ovarian response. The antral follicular count, anti-Mullerian hormone concentration, and induction length were found to be predictors of ovarian response. The AFC was the strongest predictor, and combination testing did not improve its accuracy. Thus, in settings with limited resources, the AMH is selectively used, whereas the AFC should be performed on all of our patients. Future studies are required to verify the findings about the optimal cutoff point, evaluate ovarian responses in each treatment protocol, and explore additional potential factors influencing ovarian response.

## Data Availability

The data can be obtained from the corresponding author upon a reasonable request.

## Abbreviations

AFC: antral follicle count
AMH: anti-Mullerian hormone
ANOVA: analysis of variance
AOR: adjusted odds ratio
ART: assisted reproductive technology
AUC: area under the curve
BMI: body mass index
COC: combined oral contraceptive
COS: controlled ovarian stimulation
FSH: follicle stimulating hormone
hMG: human menopausal gonadotropins
IQR: interquartile range
IVF: in vitro fertilization
LH: luteinizing hormone
PCOS: polycystic ovarian syndrome
ROC: receiver operating characteristics
SD: standard deviation
TSH: thyroid stimulating hormone

## References

[1] Zegers-Hochschild F, Adamson GD, Dyer S, Racowsky C, De Mouzon J, Sokol R (2017). The international glossary on infertility and fertility care, 2017. Hum Reprod.

[2] Mascarenhas MN, Flaxman SR, Boerma T, Vanderpoel S, Stevens GA (2012). National, regional, and global trends in infertility prevalence since 1990: a systematic analysis of 277 health surveys. PLoS Med.

[3] Legese N, Tura AK, Roba KT, Demeke H (2023). The prevalence of infertility and factors associated with infertility in Ethiopia: analysis of Ethiopian demographic and Health Survey (EDHS). PLoS ONE.

[4] Nelson SM, Telfer EE, Anderson RA (2013). The ageing ovary and uterus: new biological insights. Hum Reprod Update.

[5] Broekmans F, Kwee J, Hendriks D, Mol B, Lambalk C (2006). A systematic review of tests predicting ovarian reserve and IVF outcome. Hum Reprod Update.

[6] Fauser B, Diedrich K, Devroey P, Evian Annual Reproduction Workshop Group 2007 (2008). Predictors of ovarian response: progress towards individualized treatment in ovulation induction and ovarian stimulation. Hum Reprod Update.

[7] Sunkara SK, Rittenberg V, Raine-Fenning N, Bhattacharya S, Zamora J, Coomarasamy A (2011). Association between the number of eggs and live birth in IVF treatment: an analysis of 400 135 treatment cycles. Hum Reprod.

[8] Arslan M, Bocca S, Mirkin S, Barroso G, Stadtmauer L, Oehninger S (2005). Controlled ovarian hyperstimulation protocols for in vitro fertilization: two decades of experience after the birth of Elizabeth Carr. Fertil Steril.

[9] Fleming R, Seifer DB, Frattarelli JL, Ruman J (2015). Assessing ovarian response: antral follicle count versus anti-Müllerian hormone. Reprod Biomed Online.

[10] Himabindu Y, Sriharibabu M, Gopinathan K, Satish U, Louis TF, Gopinath P (2013). Anti-mullerian hormone and antral follicle count as predictors of ovarian response in assisted reproduction. J Hum Reproductive Sci.

[11] Hendriks DJ, Mol B-WJ, Bancsi LF, Te Velde ER, Broekmans FJ (2005). Antral follicle count in the prediction of poor ovarian response and pregnancy after in vitro fertilization: a meta-analysis and comparison with basal follicle-stimulating hormone level. Fertil Steril.

[12] Jayaprakasan K, Campbell B, Hopkisson J, Johnson I, Raine-Fenning N (2010). A prospective, comparative analysis of anti-Müllerian hormone, inhibin-B, and three-dimensional ultrasound determinants of ovarian reserve in the prediction of poor response to controlled ovarian stimulation. Fertil Steril.

[13] Oudendijk J, Yarde F, Eijkemans M, Broekmans F, Broer S (2012). The poor responder in IVF: is the prognosis always poor? A systematic review. Hum Reprod Update.

[14] Technology SfAR. American Society for Reproductive Medicine (2007). Assisted reproductive technology in the United States: 2001 results generated from the American Society for Reproductive Medicine. Fertil Steril.

[15] Hendriks DJ, Kwee J, Mol BW, te Velde ER, Broekmans FJ (2007). Ultrasonography as a tool for the prediction of outcome in IVF patients: a comparative meta-analysis of ovarian volume and antral follicle count. Fertil Steril.

[16] Biljan M, Buckett W, Dean N, Phillips S, Tan S (2000). The outcome of IVF–embryo transfer treatment in patients who develop three follicles or less. Hum Reprod.

[17] De Sutter P, Dhont M (2003). Poor response after hormonal stimulation for in vitro fertilization is not related to ovarian aging. Fertil Steril.

[18] Ulug U, Ben-Shlomo I, Turan E, Erden HF, Akman MA, Bahceci M (2003). Conception rates following assisted reproduction in poor responder patients: a retrospective study in 300 consecutive cycles. Reprod Biomed Online.

[19] van Rooij IA, Bancsi LF, Broekmans FJ, Looman CW, Habbema JDF, te Velde ER (2003). Women older than 40 years of age and those with elevated follicle-stimulating hormone levels differ in poor response rate and embryo quality in in vitro fertilization. Fertil Steril.

[20] Galey-Fontaine J, Cédrin-Durnerin I, Chaïbi R, Massin N, Hugues J-N (2005). Age and ovarian reserve are distinct predictive factors of cycle outcome in low responders. Reprod Biomed Online.

[21] Inge GB, Brinsden PR, Elder KT (2005). Oocyte number per live birth in IVF: were Steptoe and Edwards less wasteful?. Hum Reprod.

[22] Saldeen P, Källen K, Sundström P (2007). The probability of successful IVF outcome after poor ovarian response. Acta Obstet Gynecol Scand.

[23] Zhen X, Qiao J, Li R, Wang L, Liu P (2008). The clinical analysis of poor ovarian response in in-vitro-fertilization embryo-transfer among Chinese couples. J Assist Reprod Genet.

[24] Orvieto R, Meltcer S, Nahum R, Rabinson J, Anteby EY, Ashkenazi J (2009). The influence of body mass index on in vitro fertilization outcome. Int J Gynecol Obstet.

[25] Badawy A, Wageah A, El Gharib M, Osman EE (2011). Prediction and diagnosis of poor ovarian response: the dilemma. J Reprod Infertil.

[26] Hansen KR, Hodnett GM, Knowlton N, Craig LB (2011). Correlation of ovarian reserve tests with histologically determined primordial follicle number. Fertil Steril.

[27] Scheffer GJ, Broekmans FJ, Dorland M, Habbema JD, Looman CW, te Velde ER (1999). Antral follicle counts by transvaginal ultrasonography are related to age in women with proven natural fertility. Fertil Steril.

[28] Abebe DB. https://sphmmc.edu.et/obgyn/reproductive-endocrinology-and-infertilty/ = [cited January 2018 August 2023].

[29] Ficicioglu C, Cenksoy PO, Yildirim G, Kaspar C (2014). Which cut-off value of serum anti-Müllerian hormone level can predict poor ovarian reserve, poor ovarian response to stimulation and in vitro fertilization success? A prospective data analysis. Gynecol Endocrinol.

[30] Ferraretti A, La Marca A, Fauser B, Tarlatzis B, Nargund G, Gianaroli L (2011). ESHRE consensus on the definition of ‘poor response’to ovarian stimulation for in vitro fertilization: the Bologna criteria. Hum Reprod.

[31] Muttukrishna S, McGarrigle H, Wakim R, Khadum I, Ranieri D, Serhal P (2005). Antral follicle count, anti-mullerian hormone and inhibin B: predictors of ovarian response in assisted reproductive technology?. BJOG: Int J Obstet Gynecol.

[32] Hassan FI, El Sheikh WA, Hablas WR, Abou Shama DE (2018). Comparative study of anti-mullerian hormone, inhibin-b, and three-dimensional ultrasound determinants of ovarian reserve in patients undergoing icsi. Egypt J Hosp Med.

[33] Padhy N, Gupta S, Mahla A, Latha M, Varma T (2010). Demographic characteristics and clinical profile of poor responders in IVF / ICSI: a comparative study. J Hum Reprod Sci.

[34] Committee ASfRMP (2015). Testing and interpreting measure of ovarian reserve: a committee opinion. Fertil Steril.

[35] Anckaert E, Smitz J, Schiettecatte J, Klein BM, Arce J-C (2012). The value of anti-Müllerian hormone measurement in the long GnRH agonist protocol: association with ovarian response and gonadotrophin-dose adjustments. Hum Reprod.

[36] Li R, Gong F, Zhu Y, Fang W, Yang J, Liu J (2016). Anti-Müllerian hormone for prediction of ovarian response in Chinese infertile women undergoing IVF/ICSI cycles: a prospective, multi-centre, observational study. Reprod Biomed Online.

[37] Hamdine O, Eijkemans M, Lentjes E, Torrance H, Macklon N, Fauser B (2015). Ovarian response prediction in GnRH antagonist treatment for IVF using anti-Müllerian hormone. Hum Reprod.

[38] Xu H, Zeng L, Yang R, Feng Y, Li R, Qiao J (2017). Retrospective cohort study: AMH is the best ovarian reserve markers in predicting ovarian response but has unfavorable value in predicting clinical pregnancy in GnRH antagonist protocol. Arch Gynecol Obstet.

[39] Li HWR, Lee VCY, Lau EYL, Yeung WSB, Ho PC, Ng EHY (2014). Ovarian response and cumulative live birth rate of women undergoing in-vitro fertilisation who had discordant anti-mullerian hormone and antral follicle count measurements: a retrospective study. PLoS ONE.

[40] Gibreel A, Maheshwari A, Bhattacharya S, Johnson NP (2009). Ultrasound tests of ovarian reserve; a systematic review of accuracy in predicting fertility outcomes. Hum Fertility.

[41] Arce J, La Marca A, Klein B, Andersen A, Fleming R (2013). Antimüllerian hormone in GnRH antagonist cycles: prediction of ovarian response and cumulative treatment outcome in good prognosis patients. Fertil Steril.

[42] Broer S, Dólleman M, Opmeer B, Fauser B, Mol B, Broekmans F (2011). AMH and AFC as predictors of excessive response in controlled ovarian hyperstimulation: a meta-analysis. Hum Reprod Update.

[43] Maheshwari A, Gibreel A, Bhattacharya S, Johnson N (2009). Dynamic tests of ovarian reserve: a systematic review of diagnostic accuracy. Reprod Biomed Online.

[44] Huang J, Lin J, Gao H, Wang Y, Zhu X, Lu X (2019). Anti-müllerian hormone for the prediction of ovarian response in progestin-primed ovarian stimulation protocol for IVF. Front Endocrinol.

[45] Nelson SM, Klein BM, Arce J-C (2015). Comparison of antimüllerian hormone levels and antral follicle count as predictor of ovarian response to controlled ovarian stimulation in good-prognosis patients at individual fertility clinics in two multicenter trials. Fertil Steril.

[46] Elgindy EA, El-Haieg DO, El-Sebaey A (2008). Anti-Müllerian hormone: correlation of early follicular, ovulatory and midluteal levels with ovarian response and cycle outcome in intracytoplasmic sperm injection patients. Fertil Steril.

[47] Lekamge DN, Barry M, Kolo M, Lane M, Gilchrist RB, Tremellen KP (2007). Anti-Müllerian hormone as a predictor of IVF outcome. Reprod Biomed Online.

[48] Van Rooij I, Broekmans F, Te Velde E, Fauser B, Bancsi L, Jong F (2002). Serum anti-Müllerian hormone levels: a novel measure of ovarian reserve. Hum Reprod.

[49] Andrisani A, Marin L, Ragazzi E, Donà G, Bordin L, Dessole F (2019). Is corifollitropin alfa effective in controlled ovarian stimulation among all poor ovarian responders? A retrospective comparative study. Gynecol Endocrinol.

[50] Brodin T, Hadziosmanovic N, Berglund L, Olovsson M, Holte J (2013). Antimüllerian hormone levels are strongly associated with live-birth rates after assisted reproduction. J Clin Endocrinol Metabolism.

[51] Rosen MP, Johnstone E, McCulloch CE, Schuh-Huerta SM, Sternfeld B, Reijo-Pera RA (2012). A characterization of the relationship of ovarian reserve markers with age. Fertil Steril.

[52] Tsakos E, Tolikas A, Daniilidis A, Asimakopoulos B (2014). Predictive value of anti-müllerian hormone, follicle-stimulating hormone and antral follicle count on the outcome of ovarian stimulation in women following GnRH-antagonist protocol for IVF/ET. Arch Gynecol Obstet.

[53] DEHGHANI FR, Tayebi N, Asgharnia M. Serum level of anti-mullerian hormone in early follicular phase as a predictor of ovarian reserve and pregnancy outcome in assisted reproductive technology cycles. 2008. 18588367

[54] Zheng H, Chen S, Du H, Ling J, Wu Y, Liu H (2017). Ovarian response prediction in controlled ovarian stimulation for IVF using anti-Müllerian hormone in Chinese women: a retrospective cohort study. Med (Baltim).

[55] Nelson SM, Yates RW, Fleming R (2007). Serum anti-Müllerian hormone and FSH: prediction of live birth and extremes of response in stimulated cycles—implications for individualization of therapy. Hum Reprod.

[56] Bancsi LF, Broekmans FJ, Eijkemans MJ, de Jong FH, Habbema JDF, te Velde ER (2002). Predictors of poor ovarian response in in vitro fertilization: a prospective study comparing basal markers of ovarian reserve. Fertil Steril.

[57] Ng EH, Tang OS, Ho PC (2000). The significance of the number of antral follicles prior to stimulation in predicting ovarian responses in an IVF programme. Hum Reprod (Oxford England).

[58] Frattarelli JL, Lauria-Costab DF, Miller BT, Bergh PA, Scott RT (2000). Basal antral follicle number and mean ovarian diameter predict cycle cancellation and ovarian responsiveness in assisted reproductive technology cycles. Fertil Steril.

[59] Conceição MAJ, Bilibio JP, Longhi LP, De Conto E (2021). Association of FSHR, LH, LHR, BMP15, GDF9, AMH, and AMHR polymorphisms with poor ovarian response in patients undergoing in vitro fertilization. JBRA Assist Reprod.

